# Autism spectrum disorders: an updated guide for genetic counseling

**DOI:** 10.1590/S1679-45082017RB4020

**Published:** 2017

**Authors:** Karina Griesi-Oliveira, Andréa Laurato Sertié

**Affiliations:** 1 Hospital Israelita Albert Einstein, São Paulo, SP, Brazil.

**Keywords:** Autistic disorder, Genetic counseling, Microarray analysis, Genetic testing, Pathology, molecular

## Abstract

Autism spectrum disorder is a complex and genetically heterogeneous disorder, which has hampered the identification of the etiological factors in each patient and, consequently, the genetic counseling for families at risk. However, in the last decades, the remarkable advances in the knowledge of genetic aspects of autism based on genetic and molecular research, as well as the development of new molecular diagnostic tools, have substantially changed this scenario. Nowadays, it is estimated that using the currently available molecular tests, a potential underlying genetic cause can be identified in nearly 25% of cases. Combined with clinical assessment, prenatal history evaluation and investigation of other physiological aspects, an etiological explanation for the disease can be found for approximately 30 to 40% of patients. Therefore, in view of the current knowledge about the genetic architecture of autism spectrum disorder, which has contributed for a more precise genetic counseling, and of the potential benefits that an etiological investigation can bring to patients and families, molecular genetic investigation has become increasingly important. Here, we discuss the current view of the genetic architecture of autism spectrum disorder, and list the main associated genetic alterations, the available molecular tests and the key aspects for the genetic counseling of these families.

## RESUMO

O transtorno do espectro autista é um distúrbio complexo e geneticamente heterogêneo, o que sempre dificultou a identificação de sua etiologia em cada paciente em particular e, por consequência, o aconselhamento genético das famílias. Porém, nas últimas décadas, o acúmulo crescente de conhecimento oriundo das pesquisas sobre os aspectos genéticos e moleculares desta doença, assim como o desenvolvimento de novas ferramentas de diagnóstico molecular, tem mudado este cenário de forma substancial. Atualmente, estima-se que, por meio de testes moleculares, é possível detectar uma alteração genética potencialmente causal em cerca de 25% dos casos. Considerando-se também a avaliação clínica, a história pré-natal e a investigação de outros aspectos fisiológicos, pode-se atribuir uma etiologia para aproximadamente 30 a 40% dos pacientes. Assim, em vista do conhecimento atual sobre a arquitetura genética do transtorno do espectro autista, que tem tornado o aconselhamento genético cada vez mais preciso, e dos potenciais benefícios que a investigação etiológica pode trazer aos pacientes e familiares, tornam-se cada vez mais importantes os testes genéticos moleculares. Apresentamos aqui uma breve discussão sobre a visão atual da arquitetura genética dos transtornos do espectro autista, listando as principais alterações genéticas associadas, os testes moleculares disponíveis e os principais aspectos a se considerar para o aconselhamento genético destas famílias.

## INTRODUCTION

Autism spectrum disorder (ASD) is a group of neurodevelopmental early onset disorders, characterized by impairment in social and communicative skills and stereotyped behavior.^1^ Although defined by this core of symptoms, the phenotype of ASD patients is extremely variable, ranging from individuals with severe intellectual disability (ID) and very low performance on adaptive behavior skills, to individuals with normal intelligence quotient (IQ) that can live an independent life. These individuals can also present a series of other comorbidities, such as hyperactivity, sleeping and gastrointestinal problems, and epilepsy.^2^ Autism spectrum disorder has been estimated to affect 1% of the population and is four times more prevalent among males than among females.^3^

Although environmental factors, such as infections or the use of certain drugs during pregnancy, are thought to play a role in its etiology, heritability of ASD has been estimated to be around 50 to 90%, showing the relevance of genetic factors in the pathogenesis of the disease.^4,5^ The understanding of the genetic aspects of a disease provides valuable information on recurrence risks, prognostics and possible therapeutic interventions. In this regard, the great efforts spent in the last decades for a better comprehension of genetic factors associated with ASD have greatly improved the diagnostic yields and genetic counseling for this disease. Here, we discuss the current view of the genetic architecture of ASD, pointing out the guidelines for molecular screening and genetic counseling for ASD patients.

### Genetic architecture of autism spectrum disorder

Autism spectrum disorder is considered a complex and genetically heterogeneous disease, since it can present different patterns of inheritance and underlying genetic variants. In order to understand the currently defined genetic architecture of ASD, it is important to consider the epidemiological and evolutionary aspects, as well as the whole body of knowledge regarding molecular alterations related to the disease. First, we should consider a primordial evolutionary rule that influence the frequency of genetic variants in the population: if a certain genetic variant has a damaging effect to the organism, and negatively affect the fitness of the individual (its reproductive chances), this variant tends to have a low frequency in the population, since it is not passed down through the generations. Indeed, this is what is found for most monogenic diseases: they are usually rare in the population as it is the frequency of their causing alleles. Following this assumption, if a disease that reduces fitness is common in the population, it is unlikely that it is caused by a unique variant with very deleterious functional impact. Instead, it is hypothesized that common diseases with genetic basis have a polygenic or multifactorial (genes plus environmental factors) model of inheritance: they are caused by the inheritance of a combination of genetic variants, each of them conferring a low risk for the disease. Since the phenotypic impact of each variant is low, if an individual carries just a few or some of them, he/she will not have the disease and the variants can be continuously passed down from generation to generation, thus becoming common in the population. As a consequence, the chance of an individual inheriting enough number of these low-risk variants to cause the disease is not so rare. A deeper discussion on this topic can be found in El-Fishawy et al.^6^

**Figure 1:**
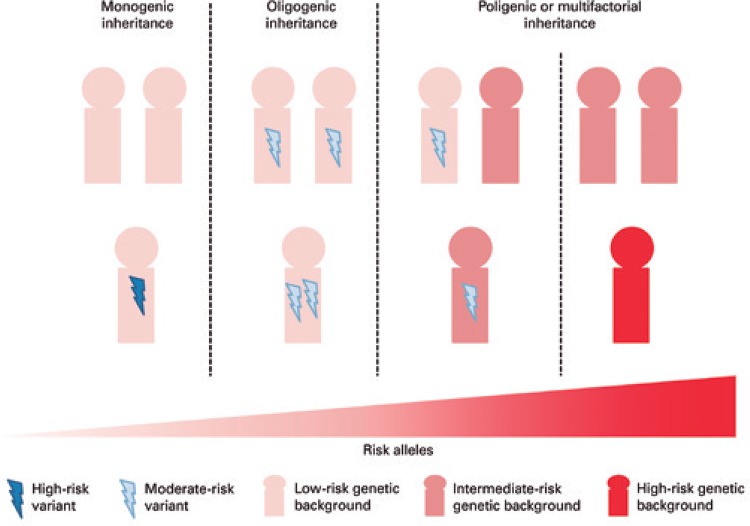
Inheritance patterns of autism spectrum disorder

Adapted from: Bourgeron T. From the genetic marachitere to synaptic plasticity in autism spectrum disorder. Nat Rev Neurosci. 2015;16(9):551-63. Review.

Based on these concepts, a polygenic or multifactoral pattern of inheritance used to be considered the inheritance pattern accounting for most cases of ASD. However, along the years, a considerable number of ASD individuals were found to harbor rare mutations, which have a damaging effect on neuronal development probably sufficient to be responsible for the disease by themselves.^7-10^ Also, in some families, a same rare potentially damaging genetic variant is shared among affected individuals, although also present in non-affected family members, resembling a monogenic pattern of inheritance with incomplete penetrance of the phenotype. Since then, the inheritance patterns of ASD have been reviewed and, currently, the interplay between common and rare variants seems more likely to fit in all these findings and explain the underlying genetic architecture of the disorder (Figure 1). Therefore, in this scenario, part of the cases would be caused by a high load of low-risk common variants that, together, can trigger disease development. Another set of the cases would harbor an intermediate load of low-risk common variants and would develop ASD if receiving a rare variant with moderate risk. Also, there are cases with a low burden of low-risk variants that would develop the disease if receiving a few moderate-risk variants. In all these situations, the risk of recurrence of ASD in the family is higher than in the general population, since there are risk alleles segregating in the family. Finally, ASD could also be caused by a unique rare damaging mutation. These high-risk mutations are usually associated with high penetrance and are *de novo* events. In this case, the recurrence risk in the family would be the same of the general population (except for germline mutations). Although common variants might contribute largely to ASD risk, they are difficult to be recognized, as they are associated with subtle effects, and remain mostly unknown.^11,12^ Therefore, much of our knowledge about the genes underlying ASD has come from studies that identified variants with moderate to high risk. It has been estimated that variants in more than 400 genes and several copy number variations (CNVs) (deletions and duplications events) can represent high to moderate risk variants for the disease.^13-15^ A full accurate and updated list of ASD risk genes, including scientific evidence that base their involvement in the disease, is available at Sfari Gene database (https://gene.sfari.org/autdb/Welcome.do). Reported cases of CNV in ASD individuals can be found at DECIPHER database (https://decipher.sanger.ac.uk/). Importantly, none of these genetic alterations accounts individually for more than 1% of total cases of ASD.^16^

Low and high-risk variants can contribute to ASD etiology. A variant harboring a high risk is enough to cause the disease alone. In such situation, usually the variant has risen as a *de novo* mutation, and parents do not harbor risk variants, characterizing a monogenic attern of inheritance. In a second situation, characterizing an oligogenic pattern of inheritance, parents can harbor moderate-risk variants that can be inherited by the child, leading to the disease. A polygenic or multifactorial patter of inheritance would be the cases of inheritance of a moderate load of low-risk variants plus a moderate-risk variant, or a high load of low-risk variants.

### Molecular tests and genetic counseling in autism spectrum disorder

Genetic counselling for ASD involves (1) explaining the genetic aspects of the disease to parents; (2) clinical evaluation of the patient and assessment of family history; (3) discussing genetic testing options; (4) interpreting the results; (5) informing about medical treatments and prognosis; and (6) reporting the recurrence risks to parents and eventually to the own patient. Next, we discuss each of these steps briefly.

First, it should be made clear to the family that ASD is a complex genetically heterogeneous disorder, which makes the genetic counseling very challenging. Based on the current knowledge, a genetic variant can only be assigned as a major etiologic cause of ASD if it is associated with a high risk for the development of the disorder, while sets of common low-risk variants that would lead to polygenic or multifactorial forms of ASD are still not recognizable. Therefore, parents should be aware that molecular testing will be able to provide an assertive molecular diagnosis in only a small proportion of cases.

**Figure 2:**
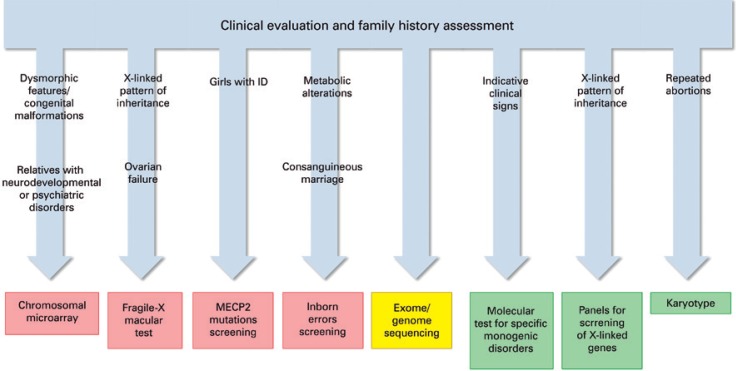
Genetic counseling in autism spectrum disorder

For the majority of ASD cases, there are no clinical signs indicating a specific genetic alteration. However, ASD can be part of the symptoms of a couple of monogenic and metabolic disorders (Table 1). Thus, a careful clinical evaluation of the patient and assessment of family history, which can give some insights on the pattern of inheritance, can improve the diagnostic yield and the choice of appropriated molecular tests to be applied in each particular case (Figure 2).

**Table 1:** Selected monogenic syndromes* associated with autism spectrum disorder and corresponding genes

Syndrome	Mutated gene
Fragile-X syndrome	*FMR1*
Rett syndrome	*MECP2*
Cowden syndrome	*PTEN*
Neurofibromatosis	*NF1*
Tuberous sclerosis	*TSC1/2*
CHARGE syndrome	*CHD7*
Sotos syndrome	*NSD1*
Beckwith-Wiedemann syndrome/Silver-Russel syndrome	*IGF2 (11p15)*
Timothy syndrome	*CACNA1C*
Noonan syndrome	*PTPN11*
Angelman syndrome	*UBE3A (15q11-q13)*
Rubinstein-Taybi syndrome	*CREBBP*
Smith-Magenis syndrome/Potocki-Lupski syndrome	*RAI1*
Velocardiofacial/DiGeorge syndrome	22q11 deletion
Phelan-McDermid syndrome	22q13 deletion
Duchenne muscular distrophy	*DMD*
Cornelia de Lange syndrome	*SMC1A*

Source: Betancur C. Etiological heterogeneity in autism spectrum disorders: more than 100 genetic and genomic disorders and still counting. Brain Res. 2011;1380:42-77.Review.(14)

*For a complete list of monogenic syndromes associated to autism spectrum disorder.

The first important step in genetic counseling is the clinical evaluation of the patient and the assessment of family history. This can provide valuable information that can direct molecular test to a more appropriated choice. As a general rule, chromosomal microarray, fragile X test for males, MECP2 mutation screening for females and inborn errors screening should be performed for all patients diagnosed with ASD (red). Particular situations can direct the choice specifically to one of these tools. As a second-tier diagnostic tool, exome or genome sequencing can be applied (yellow). Characteristic clinical signs, pattern of inheritance or history of repeated abortions can be indicative of specific genetic alterations that can be screened with more specific molecular tools (green).

As a general rule, it is currently recommended that all ASD patients should be screened for CNV through chromosomal microarray analysis,^17^ since it is estimated that approximately 10% of the patients display a clinically significant CNV.^18^ Particularly, patients presenting with micro/macrocephaly, seizures, dysmorphic features, congenital malformations and family history of other psychiatric and neurodevelopmental disorders are found to have higher rates of clinically significant CNV.^17^ The most common CNV found in ASD patients are located at 15q11-13, 16p11 and 22q11-13, which altogether have an incidence of around 3 to 5%.^15^ Since karyotyping has a lower resolution than chromosomal microarray analysis, nowadays this cytogenetic testing has been indicated only when there is a suspicion of aneuploidy or a history of repeated abortions suggestive of chromosomal rearrangements.

Among the single-gene syndromes that have ASD as part of their symptoms, three deserves special attention. Due to its elevated prevalence among ASD individuals,^2^ molecular testing for fragile X syndrome should be performed for all male patients, despite the absence of any characteristic clinical feature of the syndrome.^17^ Female screening for fragile X is only recommended in cases of X-linked pattern of inheritance of undiagnosed ID, family history of fragile X syndrome or premature ovarian failure. It is also recommended that all ASD girls with ID should be tested for mutations in *MECP2* gene, responsible for Rett syndrome, since it has been estimated that 4% of female ASD patients with severe ID present damaging mutations in this gene.^17^ Finally, mutations in *PTEN* gene, which are associated with harmatoma tumor syndromes, a disease that causes macrocephaly/macrosomy and increased risk for tumorigenesis, should be screened in ASD cases with macrocephaly (head circumference above 2.5 SD of the mean), specially due to increased risk of cancer development.^17^

ASD can also be associated with metabolic disorders in a relatively small proportion of the cases. Despite the fact that metabolic disorders are mainly associated with recessive pattern of inheritance (thus being more indicative in cases of consanguineous marriage) and present clear characteristic clinical features such as seizures, neurological regression and other physiological abnormalities, it has been suggested that screening for inborn metabolic errors should be performed for all ASD patients.^17^

More recently, exome and whole-genome sequencing has become more affordable and started to be used in clinical practice. In fact, *de novo* disruptive single nucleotide variants are estimated to be found in around 8 to 20% of ASD cases.^15,19^ They are particularly enriched in ASD patients with moderate to severe intellectual deficiency in contrast to patients with normal IQ.^16^ It should be noted that, except in cases in which the potential causing variant is a rare loss-of-function mutation located in a well established candidate gene, results from exome and especially whole genome sequencing are still difficult to interpret. On the other side, it is expected that sequencing data from large cohorts of autistic individuals that are being generated by big consortiums will facilitate the interpretation of such results in a close future. Hence, next-generation sequencing technologies are still not considered as a first-tier diagnostic tool, but with new analysis approaches being developed, lowering prices and the increasing amount of knowledge being generated, it will probably become the gold standard molecular test for ASD.

Unfortunately, for the majority of ASD cases, a prognosis or a specific medical treatment/conduct based on the genetic alteration is not available. There are a few exceptions, however, such as in cases of metabolic disorders or monogenic syndromes associated with tumorigenesis and a comorbid ASD diagnosis, as harmatoma tumor syndromes, neurofibromatosis type I, and tuberous sclerosis syndrome. These examples illustrate the importance of correct diagnosis, since it can enable treatment and/or prevention of health issues.

Although chromosomal microarray analysis and next generation sequencing technologies − which have enabled the high throughput screening of the genome − have greatly improved the diagnostic yield for ASD, variants that can be assigned as the etiological factors can be identified only in around 25% of the patients.^16^ Considering the clinical phenotype and family history, combined with biochemical and molecular testing for known metabolic and monogenic ASD-related syndromes, the etiology of ASD can be determined for approximately 30 to 40% of the cases.^17^ In such situations, recurrence risks can be more assertive. However, it is worth noting that many well-known ASD related variants are associated with susceptibility to other psychiatric phenotypes or incomplete penetrance, hampering a reliable estimation of recurrence risk. In cases of ASD with no identifiable cause, recurrence risk is based on empirical observations: for a couple with one affected child, it is calculated to be around 3 to 10%, and it can be considered higher (~7%) if the affected child is a female, or lower (~4%) if the affected child is a male. Also, having two or more affected children, the recurrence risk rises to 33 to 50%.^5,20^

Despite of all these advances, there is still a small proportion of families with an autistic child that see a genetic counselor.^21^ Genetic counseling can greatly benefit these families since it can supply them with appropriate information, which can influence reproductive decisions and, in some cases, even the clinical conduct. Finding a biological explanation for the disease can also cause a feeling that every possible effort to help a child’s treatment have been done, bringing some kind of relief to the parents. Finally, it is believed that the rapidly evolving knowledge that has been brought by autism genetics research field will certainly contribute to the development of more accurate diagnostics and possibly of specific genetic-based treatments, making the investigation of genetic etiology for ASD children extremely important.
